# Potent Antivirals for Respiratory Syncytial Virus Detected via Peptide-Based Fusion Inhibitor Screening

**DOI:** 10.1021/acsomega.6c05253

**Published:** 2026-10-07

**Authors:** Satoshi Suzuki, Yusuke Usui, Keisuke Aoki, Haruto Amano, Yoshiki Hiramatsu, Akie Nishiyama, Koichi Hashimoto, Kazutaka Murayama, Hironori Hayashi, Stefan G. Sarafianos, Shinya Oishi, Eiichi N. Kodama

**Affiliations:** † Department of Infectious Diseases, 13101 Tohoku University Graduate School of Medicine, 2-1 Seiryo-machi, Aoba-ku, Sendai, Miyagi 980-8575, Japan; ‡ Department of Bioinformatics and Integrative Omics, National Institute of Infectious Diseases, Japan Institute for Health Security, Musashi-murayama, Tokyo 208-0011, Japan; § Laboratory of Medicinal Chemistry, 12916 Kyoto Pharmaceutical University, 1 Misasagi-Shichono-cho, Yamashina-ku, Kyoto 607-8412, Japan; ∥ Graduate School of Pharmaceutical Sciences, Kyoto University, 46-29 Yoshida-Shimo-Adachi-cho, Sakyo-ku, Kyoto 606-8501, Japan; ⊥ Division of Infectious Diseases, International Research Institute of Disaster Science, Tohoku University, 2-1 Seiryo-machi, Aoba-ku, Sendai, Miyagi 980-8575, Japan; # Department of Pediatrics, School of Medicine, 12775 Fukushima Medical University, 1 Hikariga-oka, Fukushima City, Fukushima 960-1295, Japan; ∇ Division of Biomedical Measurements and Diagnostics, Graduate School of Biomedical Engineering, 200695 Tohoku University, 2-1 Seiryo-machi, Aoba-ku, Sendai, Miyagi 980-8575, Japan; ○ Center for ViroScience and Cure, Laboratory of Biochemical Pharmacology, Department of Pediatrics, 1371 Emory University School of Medicine, Atlanta, Georgia 30322, United States; ◆ Tohoku Medical Megabank Organization, Tohoku University, 2-1 Seiryo-machi, Aoba-ku, Sendai, Miyagi 980-8575, Japan

## Abstract

The respiratory syncytial virus (RSV) causes fatal pneumonia in neonates, infants, and older adults. However, no approved antiviral drugs exist for RSV; furthermore, the currently available antibody-based therapies are expensive and do not provide complete protection against RSV infections. Thus, developing anti-RSV agents for treating RSV infections is crucial. We previously established a rational strategy for designing peptide-based fusion inhibitors targeting class I fusion proteins, which are essential for the membrane fusion of viruses and host cells. Here, we used this strategy to identify peptide-based fusion inhibitors that block the interaction between the heptad repeat 1 and 2 (HR1 and -2) regions of the RSV F protein and identified four key amino acid regions involved in the HR1–HR2 interaction. Peptides, including the key amino acid regions Vir^RS^2 and Vir^RS^8, inhibited the replication of the RSV-A2 strain. We confirmed the structure–activity relationships using crystal structure coordinates and AlphaFold2-based structural predictions. These results may advance novel RSV fusion inhibitor development.

## Graphical abstract

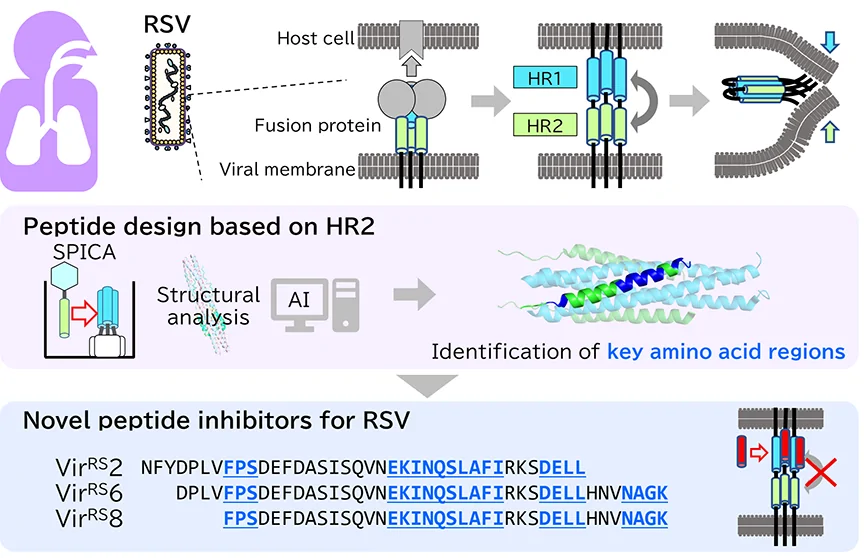

## Introduction

Respiratory syncytial virus (RSV) is an enveloped RNA virus belonging to the family *Pneumoviridae* and causes acute lower respiratory tract infections. RSV infection is a common disease in children under 2 years of age, although most infections resolve on their own within 1–2 weeks. Low-birth-weight infants and children with chronic diseases are at a risk of severe or even fatal respiratory failure.^1,2^ Additionally, older patients may experience respiratory failure due to RSV infection.^3^ In adults, the severity of RSV-associated disease is comparable to that of influenza, as evidenced by similar rates of intensive care unit admission among older patients. Specifically, the number of older patients requiring intensive care is estimated to be comparable to that of patients with influenza A viral infection.^3^

Vaccinations are used as a treatment approach, but they fail to provide complete protection against RSV infection.^4^ Furthermore, whether these vaccines provide long-term immunity remains unclear. Additional concerns remain regarding whether vaccine-resistant variants of RSV may emerge, as observed for severe acute respiratory syndrome coronavirus type 2 (SARS-CoV-2).^5,6^ Therefore, a definitive and effective treatment is required for RSV infections in individuals of all ages. However, no approved antiviral drugs exist for RSV. Currently, the monoclonal antibodies palivizumab^7^ and nirsevimab^8^ that have been approved for RSV infection prevention in high-risk infants and children are the only options available. However, these expensive antibody-based therapies do not provide complete protection against RSV infections.^7,8^

RSV enters host cells via multiple pathways including membrane fusion and endocytosis.^9–12^ The fusion (F) protein is a major RSV surface protein that plays a central role in viral entry by mediating membrane merging between the viral envelope and host cell membranes^9^ (Figure 1). Upon activation, the F protein undergoes substantial conformational rearrangement that drives the fusion process, enabling the release of the viral genome into the cytoplasm. As membrane fusion is an essential step in RSV infection regardless of the entry route, the F protein is a critical determinant of viral infectivity and a major target for antiviral intervention. Structurally, it is a Class I viral fusion protein.^13^ The complete F protein (F0) comprises two subunits: F1 and F2.^13^ The F2 subunit binds to nucleolin,^14^ epidermal growth factor receptor,^15^ or insulin-like growth factor-1 receptors^16^ of host cells. The F1 subunit mediates fusion. It contains two characteristic extravirion heptad repeats HR1 and HR2 that interact with each other to form a six-helix bundle (6HB) that facilitates the fusion of the virus with the host cell membrane^17–19^ (Figures 1 and 2). Thus, HR1–HR2 interaction inhibition prevents RSV entry into host cells.^18,20,21^ The HR1–HR2 interaction occurs outside virions and host cells; thus, inhibitors targeting this interaction are not required to be cell-permeable. For example, enfuvirtide (T-20) is an FDA-approved peptide-based HIV-1 inhibitor that successfully blocks viral entry by inhibiting the HR1–HR2 interaction in gp41.^22^ This strategy has additionally been applied for developing fusion inhibitors against viruses with class I F proteins.^23–25^

**Figure 1:**
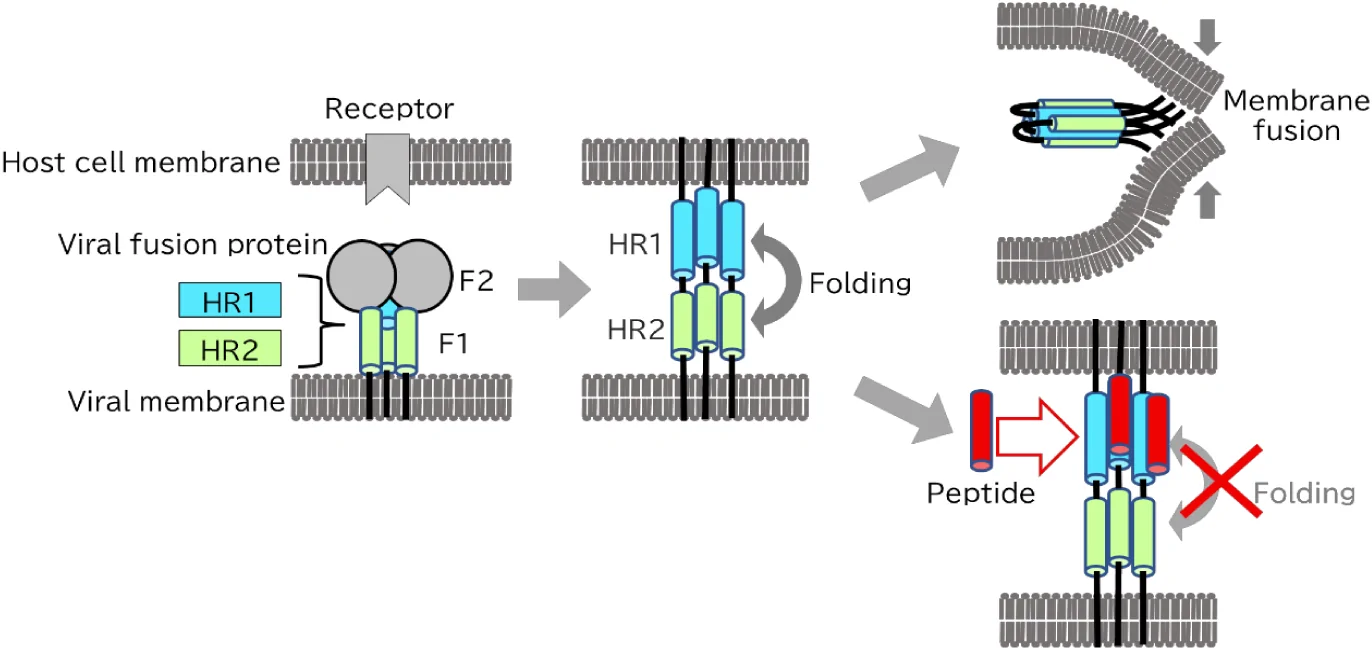
Inhibitory mechanism underlying virus–cell fusion. RSV utilizes its own membrane fusion proteins to enter host cells. After binding of the F2 protein with receptors expressed on the host cell surface, F1 acts as a bridge between the virus and host cell membrane. The N-terminal domain of F1 (the fusion peptide) is inserted into the host cell membrane. Following bridging, the HR1 and HR2 in F1 interact with each other and form a 6-helix bundle (6HB) through inter- and intramolecular folding. The 6HB formation triggers membrane fusion between the virus and the host cell. Agents blocking the HR1–HR2 interaction inhibit viral infection.

**Figure 2:**
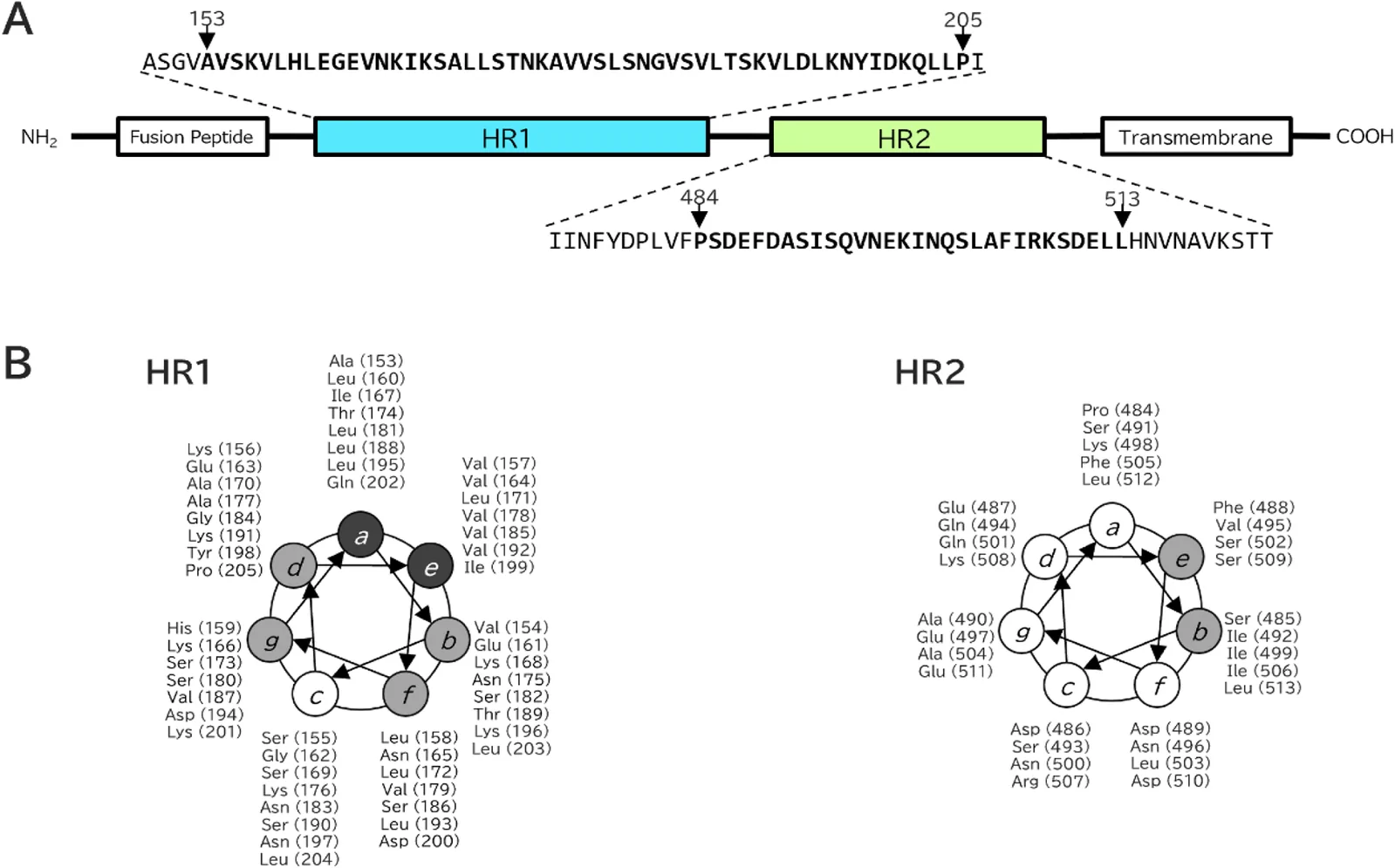
F1 protein and helical wheel of heptad repeats 1 and 2 (HR1 and HR2). (A) Representative functional domains in F1, including fusion peptide, HR1, HR2, and transmembrane domain. HR1 and HR2 sequences of the RSV-A2 strain. According to crystal structures 1G2C, 3RRR, and 5J3D in protein data bank, A153 to P205 of HR1 and P484 to L513 of HR2 form an α-helix structure (bold). (B) Helical wheels for the α-helix region of HR1 and HR2 with amino acids and sequence numbers (A2 strain) are shown. Amino acids indicated in gray (positions b, d, f, and g in HR1 and b and e in HR2) are located in the interaction interface between HR1 and HR2, and those in black (positions a and e in HR1) are involved in HR1 trimer formation.

In this study, we aimed to develop peptide-based RSV fusion inhibitors that target the HR1–HR2 interaction using a unique design strategy. We focused on identifying key amino acid regions within the HR2 segment because this region is highly conserved,^26^ and its sequence is relatively conserved^27^ across RSV strains; moreover, targeting HR1 is inefficient owing to its largely unstructured nature in solution.^28^ We identified key amino acid regions involved in the HR1–HR2 interaction of the RSV F1 protein using a rapid and convenient detection method “screening for peptide-based inhibitors containing an α-helix region” (SPICA)^29^ (Figure 3). The inhibitory effect of the synthesized peptides on the HR1–HR2 interaction was examined using SPICA^23,29–31^ and cell-based assays, which showed comparable inhibition.

**Figure 3:**
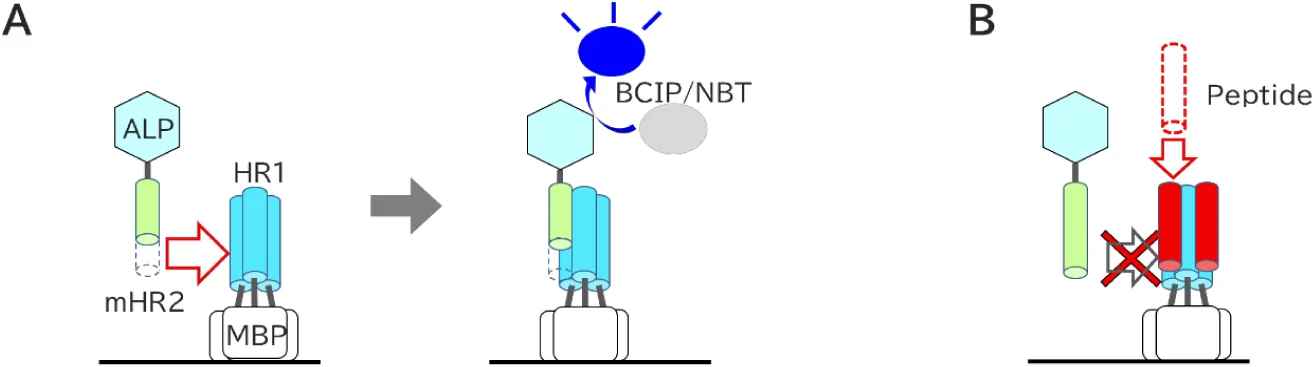
Screening for peptide-based inhibitors containing an α-helix region (SPICA). (A) Alkaline phosphatase (ALP)-fused HR2 binds to maltose-binding protein (MBP)-fused HR1 through HR1–HR2 interaction, which is detected using ALP activity based on the relative OD_630_ value. SPICA detection of the HR1–HR2 interaction with modified HR2 (mHR2). (B) Activity of a peptide fusion inhibitor was also evaluated using SPICA.

## Results

### pH Optimization of HR1–HR2 Interaction for SPICA

Certain viruses, including the influenza virus, enter host cells through endocytosis and require an acidic endosomal environment to trigger conformational changes in their fusion proteins, thereby enabling membrane fusion.^32,33^ Therefore, we optimized the pH of SPICA to detect HR1–HR2 interactions. We examined the effect of buffers of various pH levels on the interaction between full-length HR1 and full-length HR2 using 50 nM-coated maltose-binding protein (MBP)-fused HR1 (MBP–HR1) and 10 nM alkaline phosphatase (ALP)-fused HR2 (ALP–HR2) in the A2 strain (Figure S1). The full-length HR1 and HR2 regions are 58 and 50 amino acids in length, respectively.^17^ ALP activity correlated well with the HR1–HR2 interaction at 50 nM plate-coated MBP–HR1 and 10 nM ALP–HR2. The activity was the highest in buffer pH 6.0, which was chosen as the optimal pH for the HR1–HR2 interaction in SPICA.

### Key Amino Acid Regions Involved in HR1–HR2 Interaction Identified via Truncation

We identified key amino acid regions or residues involved in the HR1–HR2 interaction by fusing ALP with various lengths of the HR2 region (modified HR2; mHR2) of the A2 strain with N- or C-terminal truncation to construct full-length HR2 (Figure 4). ALP–mHR2 in reaction buffer containing 100 mM NaCl, 2 mM MgCl_2_, and 100 mM MES (pH 6.0) was serially diluted to detect HR1–HR2 interactions. The p*K*_d_ values for the interactions between each ALP–mHR2 and MBP–HR1 segment were determined and are shown as mean ± standard deviations. ALP-fused C45 ^19^ is a truncated HR2 lacking two and three residues at the N- and C-termini, respectively. It showed enhanced interactions with HR1 than with ALP–HR2. Therefore, we selected the C45 sequence as the reference for comparison with other mHR2 peptides.

**Figure 4:**
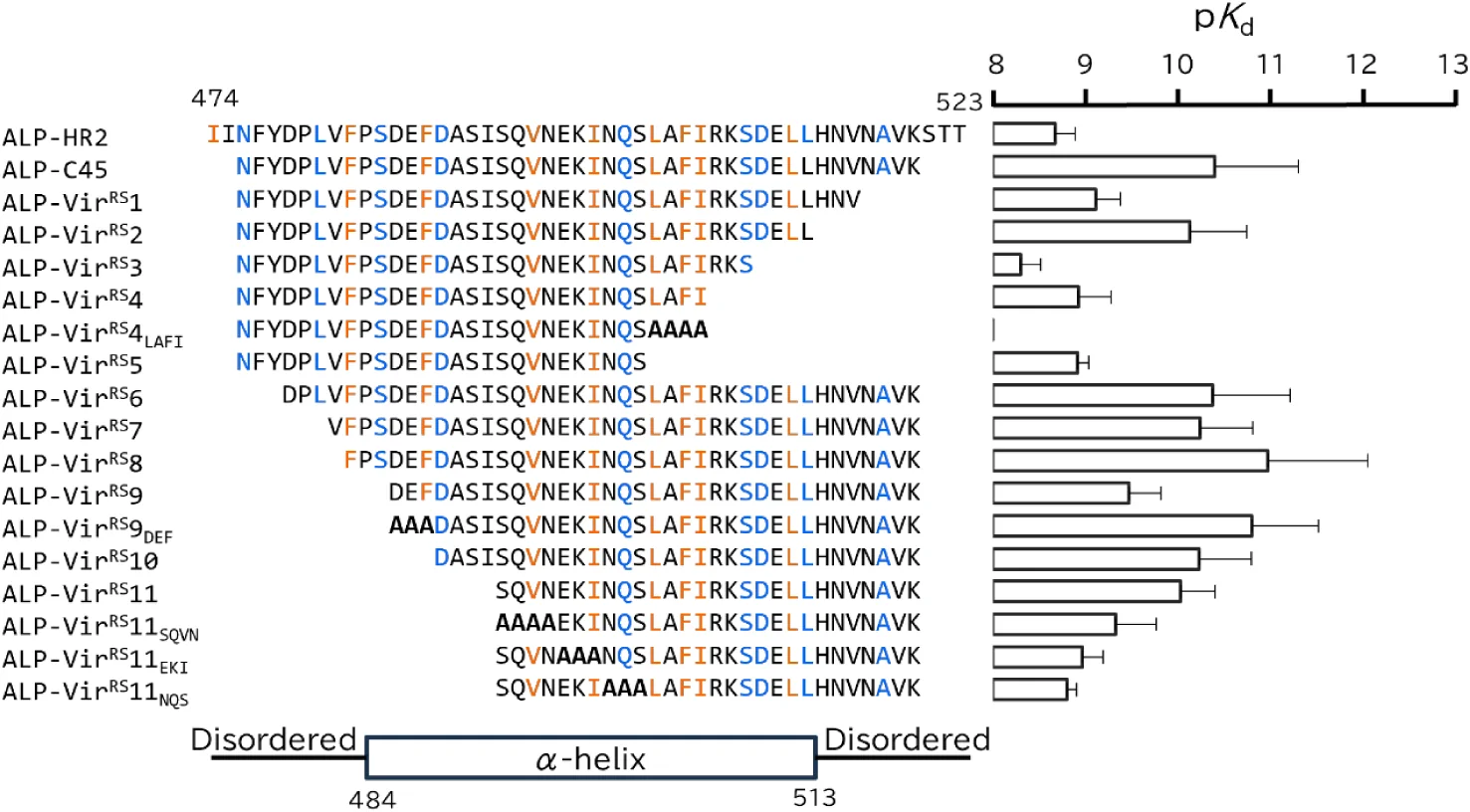
Interaction of RSV-A2 HR1–mHR2 determined using SPICA. The HR2 sequence of the A2 strain and the control peptide C45 are shown. mHR2 peptides were derived from HR2 either by partial truncation to identify functional binding regions or by alanine substitution of selected residues to assess the contribution of individual amino acids while preserving peptide length. Blue- and orange-colored amino acids indicate residues that form hydrogen bonds and hydrophobic interactions with HR1, respectively, based on previously reported crystal structures (PDB IDs: 1G2C and 3RRR). Error bars represent 95% confidence intervals.

Observing the p*K*_d_ values among C-terminus-truncated C45 derivatives (ALP-Vir^RS^1 to ALP-Vir^RS^5), we found that ALP-Vir^RS^1 without NAVK (517–520) from C45 showed lower interaction compared with that of ALP-C45, whereas ALP-Vir^RS^2 without HNV (514–516) from ALP-Vir^RS^1 restored the interaction. The truncated peptides ALP-Vir^RS^3, ALP-Vir^RS^4, and ALP-Vir^RS^5 lacking DELL (510–513), RKSDELL (507–513), and LAFIRKSDELL (503–513), respectively, showed further decreased interactions. These results indicate that the C-terminal DELL (510–513) in HR2 plays an important role in the HR1–HR2 interaction, whereas HNV (514–516) somewhat hindered the interaction, but its effect was restored by NAVK (517–520).

In contrast to the effects of C-terminal truncation, the N-terminal-truncated C45 derivatives (ALP-Vir^RS^6 to ALP-Vir^RS^11) showed comparable interactions with ALP–C45. ALP-Vir^RS^8 lacking NFYDPLV (476 to 482) showed a somewhat increased interaction, indicating that these amino acids may play a role in interfering with the HR1–HR2 interaction. However, FPS (483–485) truncation reduced this interaction. DEF (486–488)- and DEFDASI (486–492)-truncated ALP-Vir^RS^10 and ALP-Vir^RS^11, respectively, showed comparable interactions with ALP–C45. These results suggest that compared with the C-terminal region, the N-terminal region of NFYDPLVFPSDEFDASI (476–492) contributed lesser to the HR1–HR2 interaction, with the exception of FPS (483–485).

### Alanine Substitutions for Identifying Key Amino Acid Regions in HR2

As RSV-A2 HR2 contains an α-helix structure from P484 to L513 (PDB IDs: 1G2C,^19^ 3RRR,^34^ and 5J3D^35^), and its length may affect the HR1–HR2 interaction, we examined which amino acid side chains play a pivotal role in the HR1–HR2 interaction using SPICA via alanine substitutions^36^ by selectively removing side-chain functionalities while largely preserving the α-helical backbone conformation (Figure 4). As described earlier, we examined the roles of DEF (486–488), SQVN (493–496), EKI (497–499), NQS (500–502), and LAFI (503–506) in HR1–HR2 interaction. C-terminal substitution of LAFI (503–506) in ALP-Vir^RS^4 with AAAA completely abolished this interaction, suggesting that the side chains in LAFI (503–506) play a key role in the interaction. In contrast, N-terminal substitution of DEF (486 to 488) in ALP-Vir^RS^9 with AAA enhanced the interaction, indicating that α-helicity and/or the length of the peptide itself is important. However, neither EKI (497–499) nor NQS (500–502) is likely crucial in terms of either α-helicity or length of the peptides. SQVN (493–496)-substituted ALP-Vir^RS^11_SQVN_ showed an interaction comparable to that of ALP-Vir^RS^11. These results indicate that the central EKINQS (497–502) and C-terminal LAFI (503–506) play important roles in HR1–HR2 interactions.

### Inhibitory Effects of HR2-Derived Peptides in SPICA

Based on the SPICA results, we synthesized nine representative peptides from the A2 strain HR2 region. We evaluated their inhibitory effects on the HR1–HR2 interaction in SPICA using the HR1 and HR2 sequences of the A2 strain (Table 1). The HR2 peptide exhibited potent inhibitory activity with IC_50_ values in the low picomolar range. C45 ^19^ and T112 ^37^ showed slightly reduced potency, with IC_50_ values in the subnanomolar range. Vir^RS^2 is a C45-derived peptide lacking the C-terminal HNVNAGK region (residues 514–520), and it retained inhibitory activity in the 10^–11^ M range. In contrast, Vir^RS^4, which lacks a larger C-terminal region (RKSDELLHNVNAGK; residues 507–520) from C45, showed substantially reduced activity, suggesting that the RKSDELL region (residues 507–513) contributes to inhibitory potency.

**Table 1:** Inhibitory Effects of HR2 (A2 Strain)-Derived Peptides on HR1–HR2 Interaction Assessed Using SPICA^a^

Compound and peptides		Peptide sequences and molecular formula of compound		IC_50_ (nM) [pIC_50_ ± SD]
HR2	H_2_N–	IINFYDPLVFPSDEFDASISQVNEKINQSLAFIRKSDELLHNVNAGKSTT	–CONH_2_	0.0027 [11.6 ± 1.0]
C45	H_2_N–	--NFYDPLVFPSDEFDASISQVNEKINQSLAFIRKSDELLHNVNAGK	–COOH	0.053 [10.3 ± 0.5]
T112	AcHN–	--------VFPSDEFDASISQVNEKINQSLAFIRKSDELLHNV	–CONH_2_	0.024 [10.6 ± 0.7]
Vir^RS^2	H_2_N–	--NFYDPLVFPSDEFDASISQVNEKINQSLAFIRKSDELL	–CONH_2_	0.0018 [11.7 ± 1.2]
Vir^RS^4	H_2_N–	--NFYDPLVFPSDEFDASISQVNEKINQSLAFI	–CONH_2_	2.1 × 10^3^ [5.7 ± 0.4]
Vir^RS^6	H_2_N–	-----DPLVFPSDEFDASISQVNEKINQSLAFIRKSDELLHNVNAGK	–CONH_2_	0.051 [10.3 ± 0.8]
Vir^RS^8	H_2_N–	---------FPSDEFDASISQVNEKINQSLAFIRKSDELLHNVNAGK	–CONH_2_	0.072 [10.1 ± 0.5]
Vir^RS^9	H_2_N–	------------DEFDASISQVNEKINQSLAFIRKSDELLHNVNAGK	–CONH_2_	0.088 [10.1 ± 0.7]
Vir^RS^12	AcHN–	-------LVFPSDEFDASISQVNEKINQSLAFIRKSDELLHN	–CONH_2_	0.016 [10.8 ± 0.8]
VP-14637		C_25_H_22_N_10_O_3_		2.2 × 10^4^ [4.7 ± 0.4]

a Peptides HR2, C45, and T112 and the small-molecule fusion inhibitor VP-14637 were used. Effects were estimated by using the log-transformed half-maximal inhibitory concentration (pIC_50_).

Vir^RS^6, which lacks the N-terminal region of C45 but retains the FPS, EKINQS, LAFI, DELL, and NAGK motifs, maintained its inhibitory activity in the 10^–10^ M range. Furthermore, Vir^RS^8 and Vir^RS^9, which lack DPLV (residues 479–482) and DPLVFPS (residues 479–485), respectively, exhibited inhibitory activities comparable to those of Vir^RS^6 in the 10^–10^ M range. Vir^RS^12 is another mHR2 peptide that lacks both N-terminal NFYDP (476–480) and C-terminal VNAGK (516–520) from C45, and it showed inhibitory effects in the order of 10^–10^ M. VP-14637 ^38^ is a control small-molecule fusion inhibitor, and it moderately blocked the HR1–HR2 interaction with IC_50_ values in the order of 10^–4^ M, indicating that SPICA detects small-molecule inhibitors in addition to peptides (e.g., C45). Collectively, these truncation analyses delineated specific regions within C45 that differentially contributed to the inhibitory activity.

### Inhibitory Effects of HR2-Derived Peptides in SPICA for Multiple Strains

We confirmed that SPICA may be applied to strains other than A2 (subgroup A) by examining the HR1–HR2 interaction in SPICA using MBP–HR1 and ALP–HR2 of the Long (subgroup A), CH18537 (subgroup B), and A2 strains (Figure S2). A few differences were observed in the dissociation constants of HR1–HR2 interactions in SPICA among the three strains (Figure S3). The inhibitory effects of Vir^RS^2, Vir^RS^6, Vir^RS^8, and Vir^RS^12 on the HR1–HR2 interaction were detected in each strain using SPICA (Table S1), indicating that SPICA may be applied to RSV strains other than the A2 strain.

### Antiviral Effects in Cell-Based Assay

Antiviral effects of the peptides and compounds were evaluated in a cell-based assay using the RSV-A2 strain and HEp-2 cells, with known inhibitory compounds included as positive controls (Table 2). Vir^RS^2 and Vir^RS^6 showed relatively strong antiviral effects at 0.66 and 0.86 μM, respectively, compared with those of C45. Antiviral activities of Vir^RS^8 and Vir^RS^12 were comparable to that of C45. In contrast, Vir^RS^4 and Vir^RS^9 showed no antiviral activity. The small-molecule fusion inhibitor VP-14637 and viral RNA polymerase inhibitor remdesivir^39,40^ inhibited the replication of the A2 strain at 0.00023 μM and 0.010 μM, respectively. Additionally, the peptides designed and synthesized based on the SPICA results, namely, Vir^RS^2, Vir^RS^6, and Vir^RS^8, showed potential to suppress viral infections.

**Table 2:** Antiviral Effects of HR2-Derived Peptides on RSV (A2 Strain) in Cell-Based Assays^a^

	EC_50_ (μM) [pEC_50_ ± SD]	CC_50_ (μM) [pCC_50_ ± SD]
C45	2.3 [5.6 ± 0.1]	>20 [<4.7]
Vir^RS^2	0.66 [6.2 ± 0.2]	>20 [<4.7]
Vir^RS^4	>20 [<4.7]	11 [5.0 ± 0.1]
Vir^RS^6	0.86 [6.1 ± 0.1]	>20 [<4.7]
Vir^RS^8	2.0 [5.7 ± 0.1]	>20 [<4.7]
Vir^RS^9	>20 [<4.7]	>20 [<4.7]
Vir^RS^12	1.7 [5.8 ± 0.1]	>20 [<4.7]
VP-14637	0.00023 [9.6 ± 1.3]	6.1 [5.2 ± 0.4]
Remdesivir	0.010 [8.0 ± 0.5]	4.1 [5.4 ± 0.2]

a Peptide C45, the small-molecule fusion inhibitor VP-14637, and the viral RNA polymerase inhibitor remdesivir were used. The effects and cell toxicities were estimated using log-transformed half-maximal effective concentration (pEC_50_) and log-transformed half-maximal cytotoxic concentration (pCC_50_), respectively. The maximum concentration tested was 20 μM because higher peptide concentrations required increased DMSO concentrations, which caused cytotoxicity.

## Discussion

In this study, we identified the key amino acid regions involved in HR1–HR2 interaction inhibition with truncation and alanine substitutions in HR2. HR2 peptides containing the identified key amino acid regions were synthesized, and certain HR2 peptides inhibited the HR1–HR2 interaction in SPICA and exhibited antiviral activity in a cell-based assay. Notably, these peptides successfully exerted anti-RSV activity in the submicromolar concentration range, indicating that the peptides identified using SPICA are useful for identifying candidate RSV fusion inhibitors. Additionally, we reevaluated the crystal structures using AlphaFold2 to further examine the concordant and divergent features between the results obtained using SPICA and cell-based assays.

Using the previously reported crystal structure of the membrane fusion protein of the RSV-A2 strain (PDB ID: 3RRR^34^), we investigated the four key amino acid regions (FPS, EKINQSLAFI, DELL, and NAGK) using SPICA and cell-based assays. F483, which constitutes FPS (483 to 485), forms hydrophobic interactions with both HR1 and F488, suggesting the stabilization of the N-terminal α-helix structure (Figure 5A and B). This corresponds to the previously reported pivotal roles of F483 and F488.^19,41–43^ In EKINQSLAFI (497–506), Q501 formed a hydrogen bond with S180 in HR1, and I499, L503, F505, and I506 showed hydrophobic interactions with HR1 (Figure 5C and D). In DELL (510–513), D510 or L513 formed hydrogen bonds with N175 or K168, respectively, in HR1, and L512 or L513 exhibited hydrophobic interactions with HR1 (Figure 5E). As NAGK (517–520) was not included in 3RRR, we analyzed the region using another crystal structure (PDB ID: 1G2C^19^) and confirmed that A518 formed a hydrogen bond with K168 (Figure 5F). The four key regions for the HR1–HR2 interaction were confirmed using SPICA, and the crystal structures suggested that hydrogen bonds and hydrophobic interactions in these four key regions may play a pivotal role in the HR1–HR2 interaction.

**Figure 5:**
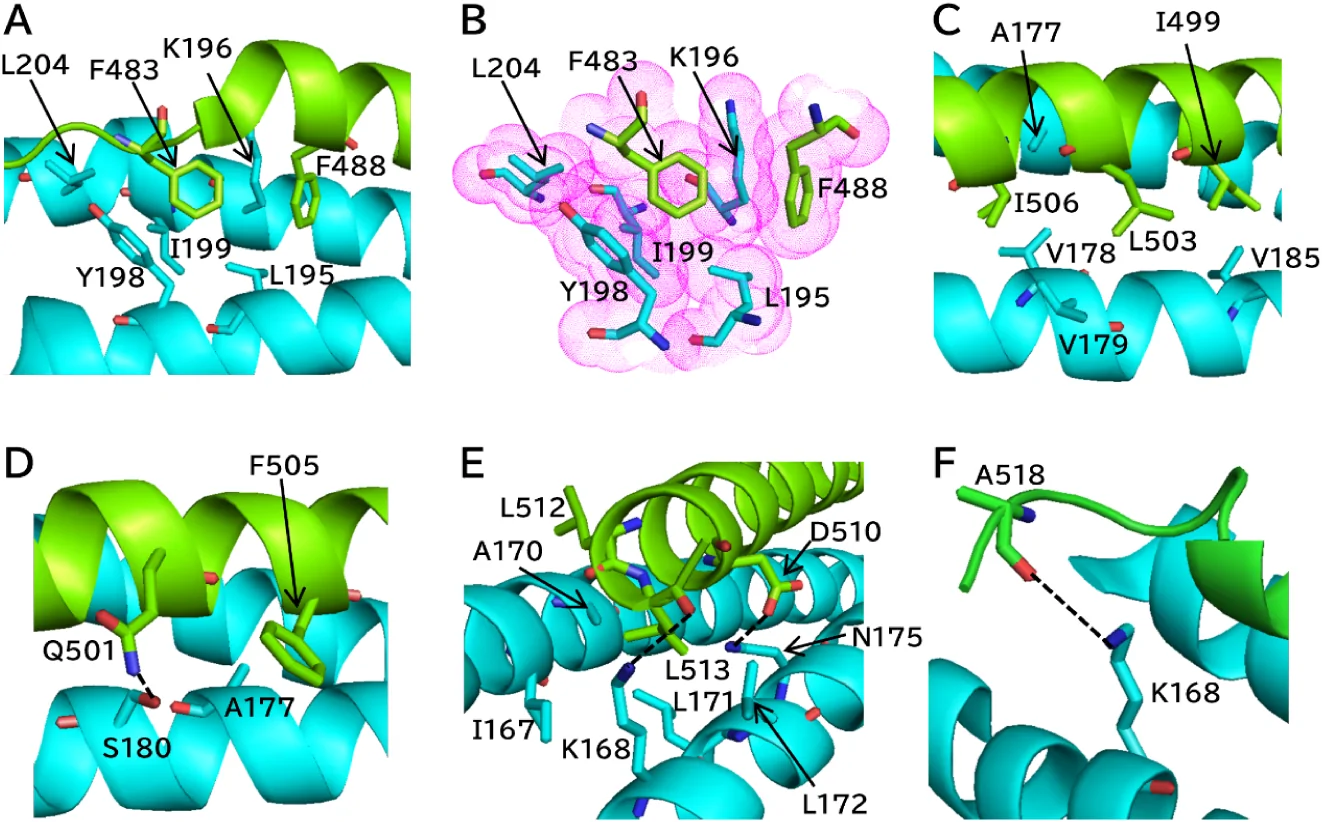
Interaction between HR1 and HR2 of RSV F protein. Crystal structures of the six-helix bundle (6HB) formed by HR1 (cyan) and HR2 (green) are shown. Amino acids involved in HR1–HR2 interactions through hydrogen bonds or hydrophobic interactions are shown as stick models. The dotted lines indicate hydrogen bonds. (A)–(E) are based on 3RRR, but NAGK (517 to 520) was not included in 3RRR. So, (F) is based on 1G2C, which contains the same amino acids as 3RRR in the identified sequences. (A)–(F) show FPS (483–485), EKINQSLAFI (497–506), DELL (510–513), and NAGK (517–520). The composition atoms in (B) are displayed in van der Waals volume.

Vir^RS^6, which showed a strong interaction in both SPICA and cell-based assays, contains DPLV (479–482). The crystal structure (PDB ID: 3RRR^34^) showed that the main chain of L481 formed a hydrogen bond with the side chain of N208, whereas the side chain of L481 engaged in hydrophobic interactions with L203, L204, I206, and V207 (Table S2). Thus, L481 may be involved in HR1–HR2 interactions. However, DPLV (479–482) was not found to be a key region. This suggests that combining peptide synthesis and cell-based assays with SPICA is useful for identifying fusion inhibitory peptides.

To further understand the strong relationship between ALP-Vir^RS^9_DEF_ with alanine substitution and ALP-Vir^RS^10 without a DEF from ALP-Vir^RS^9 in comparison with ALP-Vir^RS^9 in SPICA, we used the artificial intelligence software AlphaFold2.^44^ We found that the actual 6HB of RSV (PDB ID: 3RRR^34^) and 6HB predicted using AlphaFold2 showed high structural similarity in the superposition (root-mean-square deviation; RMSD = 0.849 Å for 201 common Cα atoms), suggesting that the AlphaFold2-predicted 6HB may be used for structural modeling.^45^ Vir^RS^9 was predicted to form an α-helix structure and the crystal structure (Figure S4A), but D486 in the predicted structure of Vir^RS^9 with a linker sequence (GGGGKL) indicated a low pLDDT score (Figure S4B). GGGG and KL were generated from an artificial insert to reduce the structural effects of the fused ALP and *Hin*dIII sites for cloning, respectively. This suggests that introduction of the linker may destabilize the structure of the N-terminus of Vir^RS^9, which showed a weak interaction in SPICA (Figure 4). Focusing on the leucine in the linker sequences (GGGGKL) of each ALP–mHR2, the leucine in ALP–Vir^RS^9 appeared to be located outside the hydrophobic pocket formed by the HR1 trimer (Figure S5A and B), whereas the leucine in the linker sequences of ALP–Vir^RS^9_DEF_ and ALP–Vir^RS^10 was predicted to be located in the hydrophobic pocket formed by the HR1 trimer and appeared to form a stable hydrophobic interaction with the surrounding amino acids of HR1 (Figure S5C–F). Hence, the leucine linker may have artificially enhanced the activities of ALP–Vir^RS^9_DEF_ and ALP–Vir^RS^10 in SPICA. Thus, computational approaches may help design linker sequences in SPICA by predicting the effect of specific amino acids on the potential structural interactions of the linkers because changes in amino acid may artificially enhance or reduce the interactions between HR1 and HR2.

Generally, peptide-based drugs with large molecular weights or instability owing to digestive enzyme cleavage are unsuitable for oral administration.^46^ However, in cases of respiratory diseases, including RSV infections, peptide-based inhibitors can be directly administered via inhalation.^47^ VP-14637 inhalation to inhibit RSV infection has been reported in a rat model.^48^ Furthermore, peptides with low cell membrane permeability may provide the advantage of low cell toxicity while influencing viral and cell-surface-targeting membrane fusion. In fact, the CC_50_ values of Vir^RS^2, Vir^RS^6, and Vir^RS^8 were higher than those of VP-14637 and remdesivir in the cell-based assay, suggesting virus-specific activity. Thus, anti-RSV peptide-based drugs may be used for inhalation administration even for outpatients or cases of short-term hospitalizations.^47^

The peptide concentrations required for antiviral activity were higher than those observed using SPICA. RSV enters host cells via endocytosis and type I membrane fusion.^10^ Fusion inhibitors may not suppress endocytosis-based entry because endocytosis does not depend on the F protein outside the host cell membranes.^10^ Other drug-escaping entry routes for viral infections may attenuate the activity of fusion inhibitors. In contrast, VP-14637 showed high activity in cell-based assays and low activity in SPICA assays. This suggests that VP-14637 possesses other antiviral mechanisms in addition to inhibiting membrane fusion, as SPICA detected only the inhibitory effects on the HR1–HR2 interaction. Although the peptides identified in this study (Vir^RS^2, Vir^RS^6, and Vir^RS^8) exhibited a higher EC_50_ than those of VP-14637 and remdesivir, this result is significant in the context of identifying drug targets. The antiviral activity of the peptides may be enhanced by modifying the side chains—such as through EK motif—in locations other than the key amino acid regions.^49^ Moreover, the relatively high CC_50_ values also suggest a favorable safety profile.

The key amino acid residues identified in this study are located within HR2 regions that are relatively conserved across RSV strains. This observation suggests that the HR2 regions involved in the HR1–HR2 interaction may be shared across diverse RSV variants. Although antiviral activity was evaluated using the RSV A2 strain in the present study, the conserved nature of the identified residues raises the possibility that peptides targeting these regions may retain activity against other circulating RSV strains,^27^ including RSV-B strains. Future studies using optimized peptide candidates and a broader panel of clinical isolates, including representative RSV-B isolates, would help determine the breadth of the antiviral activity. Notably, the present study provides a foundation for such investigations by identifying key HR2 residues through truncation and alanine-substitution analyses.

In combination with SPICA, a rapid and simple peptide design is feasible based solely on nucleotide sequence information compared with that of small-molecule fusion inhibitors. SPICA detects key amino acid regions without peptide synthesis or viral assays, thereby enabling the design of fusion-inhibitory peptide candidates. The peptides identified using SPICA, namely, Vir^RS^2 and Vir^RS^8, may be used as leads for anti-RSV peptides to inhibit HR1–HR2 interactions. As no diversity was reported among the key amino acid regions (FPS, EKINQSLAFI, and DELL) of other strains,^27^ the identified peptides may be effective on other strains, warranting further analysis. If the amino acid sequences of the peptide templates are mutated, the fusion-inhibitory peptides will remain promising templates for antiviral drugs with a quick response to SPICA and peptide synthesis with the mutated sequence. This study shows the usefulness of SPICA in combination with structural prediction using AlphaFold2 for the rational design of membrane fusion inhibiting peptides. These approaches provide a practical framework for developing RSV fusion inhibitors.

## Methods

### Antivirals and Reagents

A small-molecule RSV fusion inhibitor VP-14637 ^38^ and the viral RNA polymerase inhibitor remdesivir^39,40^ were purchased from Cayman Chemical Company (Ann Arbor, MI, USA) and Selleck Chemicals (Houston, TX, USA), respectively. Isopropyl β-D-thiogalactopyranoside (IPTG) and 3-(4,5-dimethyl-2-thiazolyl)-2,5-diphenyl-2*H*-tetrazolium bromide (MTT) were purchased from FUJIFILM Wako Chemicals (Osaka, Japan). 5-Bromo-4-chloro-3-indolyl phosphate/nitroblue tetrazolium (BCIP/NBT) was purchased from SeraCare Life Sciences (Milford, CT).

### Plasmid Construction

The HR1 region was amplified using RT-PCR with the appropriate primer sets (Table S3). RNA extracted from RSV-infected HEp-2 cells was used as the template. The amplified cDNA was cloned into the pMAL-c6T vector (New England BioLabs, Ipswich, MA) to generate MBP–HR1 (Figure S6).

The DNA fragment corresponding to the ALP coding region without the secretory signal sequence, which corresponds to the 22–471 amino acid region,^50^ was amplified from the *E. coli* JM109 genome (K12 strain, JM109, NIPPON GENE, Tokyo, Japan) using PCR with the appropriate primer set to generate ALP cDNA fused with a four-glycine linker coding DNA at the 3′-terminus (Table S3). The amplified ALP-four-glycine cDNA was cloned into the pET47b vector (pET47b-ALP) (Novagen, Billerica, MA, USA). DNA fragments of various HR2 lengths (modified HR2, mHR2) with truncation and/or alanine substitutions for HR2 were amplified as described earlier and cloned into the pET47b-ALP vector (pET47b-ALP-mHR2) (Figure S6). The cDNA coding regions for ALP and mHR2 were connected using a linker sequence encoding four glycine, a lysine, and a leucine residue derived from *Hin*dIII (GGGGKL). The HR1 and HR2 sequences of RSV were confirmed to be identical to previously deposited sequences (GenBank accession numbers KT992094, AY911262, and JX198143 for strains A2, Long, and CH18537, respectively). The primer sequences used for cloning are listed in Table S3. All cloned regions in both pMALc6T-HR1 and pET47b-ALP-mHR2 were verified through DNA sequencing.

### Protein Expression and Purification

*E. coli* BL21-Codon Plus (DE3)-RIL (Agilent Technologies, Santa Clara, CA) was transformed and incubated in a shake flask at 37 °C containing Luria broth with ampicillin for pMALc6T-HR1 and kanamycin + chloramphenicol for pET47b-ALP-mHR2. The LB culture was maintained at 37 °C until an optical density at 600 nm (OD_600_) = 0.6 was achieved. Then, the expression of each protein was induced by adding IPTG (1 mM for 4 h at 37 °C for pMALc6T-HR1 and 0.3 mM for 16 h at 25 °C for pET47b-ALP-mHR2). After incubation, the IPTG-treated *E. coli* cells were collected. To extract the expressed MBP–HR1 and ALP–mHR2 proteins, each *E. coli* pellet was resuspended in 10 mM Tris (pH 8.0) and sonicated. The cell lysates were centrifuged, and the supernatant was collected. MBP–HR1 and ALP–mHR2 were purified using Amylose Resin (New England Biolabs) and Ni-NTA Agarose (Qiagen, Venlo, Netherlands), respectively. The concentration and purity of each protein were examined using SDS-PAGE with the ChemiDoc MP imaging system (Bio-Rad, Hercules, CA, USA) and ImageJ.^51^ Bovine serum albumin (BSA; Thermo Fisher Scientific) was used as the protein standard.

### pH Optimization of HR1–HR2 Interaction in SPICA

MBP–HR1 was diluted to 50 nM with 50 mM sodium carbonate buffer (pH 9.4) and coated onto a 96-well ELISA plate (Costar 1 × 8 Stripwell high binding EIA/RIA plate, flat bottom; Corning, New York, NY) overnight at 4 °C. After washing thrice with PBS (pH 7.4) containing 0.025% Tween 20 (T-PBS), the plate was treated with 0.1% BSA in T-PBS for 2.5 h at 4 °C for blocking, followed by washing thrice with T-PBS. ALP–HR2 was serially diluted in a reaction buffer with different pH solutions containing 100 mM NaCl and 2 mM MgCl_2_. MES (100 mM) was used as a buffer solution at pH 5.6, 6.0, and 6.4. HEPES (100 mM), PBS, and Tris-HCl (10 mM) were used as buffer solutions at pH 7.0, 7.4, and 8.0, respectively. The plate was incubated for 1.5 h at 37 °C and washed thrice with the same pH buffer without ALP–HR2 containing 0.025% Tween 20. Phosphatase substrate BCIP/NBT with 1 mM Zn­(OAc)_2_ and 2 mM MgCl_2_ was added as recommended and incubated for 1 h at 37 °C. OD_630_ was measured using a microplate reader 800TS (Agilent Technologies).

### Quantification of HR1–HR2 Interaction in SPICA Using mHR2

The interaction between HR1 and mHR2 was quantified using the SPICA assay as previously described.^29^ Briefly, MBP–HR1 (50 nM) was immobilized on 96-well ELISA plates and incubated with serially diluted ALP-mHR2 proteins. After washing, bound ALP-mHR2 was detected using the BCIP/NBT substrate, and absorbance was measured at 630 nm. Relative binding activity was calculated as the ratio of the OD_630_ value at each ALP-mHR2 concentration to the maximum OD_630_ value. Dissociation constants (*K*_d_) were determined from at least three independent experiments and converted to p*K*_d_ values (−log_10_[*K*_d_ in M]).

### Peptide Synthesis

The peptides were synthesized by using standard 9-fluorenylmethoxycarbonyl (Fmoc)-based solid-phase peptide synthesis (SPPS). The peptides were purified by performing high-performance liquid chromatography (HPLC) on a preparative Cosmosil 5C18 AR-300 column (Nacalai Tesque, Inc., Kyoto, Japan) or Cosmosil 5C18 AR-II column (Nacalai Tesque, Inc., Kyoto, Japan) using a linear gradient of H_2_O/acetonitrile containing trifluoroacetic acid (0.1% or 0.05%).

### Inhibitory Effect in SPICA

MBP–HR1 (20 nM) was coated onto a 96-well ELISA plate, as described earlier. The MBP–HR1-coated plate was pretreated for 1 h at 37 °C using test peptides at various concentrations or compounds serially diluted in 100 mM NaCl, 2 mM MgCl_2_, and 100 mM MES (pH 6.0) buffer. ALP–HR2 in the same buffer was added to the plate and incubated for 1 h at 37 °C (final concentration 10 nM). After washing the plate as described earlier, BCIP/NBT was added and incubated for 1/2 h at 37 °C for Long and CH18537/A2 strains. OD_630_ was measured, and the relative OD_630_ value was calculated as the ratio of the OD_630_ value at each drug concentration to the drug-free OD_630_ value. IC_50_ values were obtained from at least three independent experiments and converted to pIC_50_ values (−log_10_[IC_50_ in M]).

### Cells and Viruses

HEp-2 cells were maintained in Dulbecco’s modified Eagle medium (DMEM) (Merck, Darmstadt, Germany) with 10% fetal bovine serum (Thermo Fisher Scientific, Waltham, MA), 100 Unit/mL penicillin, and 100 μg/mL streptomycin (Meiji Seika Pharma Co., Tokyo, Japan). The RSV-A2 strain (subgroup A) was obtained from Fukushima Medical University (Fukushima, Japan).^52^ The RSV-Long (subgroup A) and RSV-CH18537 (subgroup B) strains were obtained from ATCC (Manassas, VA, USA). All viruses were propagated in HEp-2 cells. RSV was inoculated into HEp-2 cells seeded the previous day, and the culture supernatant was collected 2 days postinfection. The supernatant was rapidly frozen in liquid nitrogen and stored at −80 °C until further use.

RSV stocks were titrated by infecting HEp-2 cells with serial dilutions of the virus, followed by microscopic observation and an MTT assay, as described previously.^53^

### Cell-Based Anti-RSV Assay

The anti-RSV effect was examined using an MTT assay.^52^ Briefly, serially diluted test peptides in the complete medium were added to flat-bottomed 96-well microtiter plates. RSV-A2 was inoculated onto HEp-2-seeded (10^4^ cells/well) plates. After 4 days, a 0.5% MTT solution (20 μL) was added to each well and incubated for 3 h at 37 °C. After incubation, 170 μL of medium was removed and replaced with 170 μL of acidified isopropanol. After complete solubilization of the formazan crystals, OD_560_ was measured. The relative OD_560_ value was calculated as the ratio of the OD_560_ value at each drug concentration to the drug-free OD_560_ value of cells without the virus. EC_50_, and CC_50_ values were obtained from at least three independent experiments, and EC_50_ values were expressed as pEC_50_ values (−log_10_[EC_50_ in M])

### Structural Modeling

Three-dimensional models of 6HB containing the HR1 and various HR2 regions were generated using AlphaFold ver.2.0 ^44^ in the multimer mode. To compare the predicted structures with the experimental model, structural superimposition was performed against the reported RSV 6HB crystal structure (PDB ID: 3RRR^34^) using Superpose in the CCP4i program package.^53^

## Supplementary Material



## Notes

Figures S1–S6 including pH optimization for the interaction of ALP–HR2 (10 nM) with MBP–HR1 (50 nM) in SPICA, amino acid regions of viral F1 protein, HR1–HR2 interaction of three strains, predicted structure of N-terminal Vir^RS^9 and HR1 by AlphaFold2, predicted structure of the linkers of ALP-Vir^RS^9, -Vir^RS^9_DEF_, and -Vir^RS^10 with HR1 by AlphaFold2, and construction of plasmids and Tables S1–S3 including inhibitory effects of the peptides for HR1–HR2 bindings determined by SPICA, interactions between amino acids in HR1 and HR2 shown by protein crystallography, and nucleotide sequence of primers for RT-PCR, sequencing, and cloning (PDF)

## Abbreviations

ALP: alkaline phosphatase
BCIP/NBT: 5-bromo-4-chloro-3-indolyl phosphate/nitroblue tetrazolium
CC_50_: 50% cytotoxic concentration
DMEM: Dulbecco’s modified Eagle medium
EC_50_: 50% effective concentration
F protein: fusion protein
HR1: heptad repeat 1
HR2: heptad repeat 2
IC_50_: 50% inhibitory concentration
IPTG: isopropyl β-D-1-thiogalactopyranoside
MBP: maltose-binding protein
MTT: 3-(4,5-dimethyl-2-thiazolyl)-2,5-diphenyl-2*H*-tetrazolium bromide
PDB: Protein Data Bank
RSV: respiratory syncytial virus
6HB: six-helix bundle
SARS-CoV-2: severe acute respiratory syndrome coronavirus 2
SPICA: screening for peptide-based inhibitors containing an α-helix
