# Supplementary material for: Potent Antivirals for Respiratory Syncytial Virus Detected via Peptide-Based Fusion Inhibitor Screening
Source: ACS Omega. 2026 Oct 7;11(41):63006–15. doi: 10.1021/acsomega.6c05253 (PMC13689203; doi:10.1021/acsomega.6c05253)
Supplement: Supplementary file 1 [file ao6c05253_si_001.pdf]

Potent antivirals for respiratory syncytial virus detected via peptide-based fusion inhibitor screening

Satoshi Suzuki[1,2], Yusuke Usui[3,4], Keisuke Aoki[3,4], Haruto Amano[3], Yoshiki Hiramatsu[1], Akie Nishiyama[1], Koichi Hashimoto[6], Kazutaka Murayama[7], Hironori Hayashi[5], Stefan G. Sarafianos[8], Shinya Oishi[3,4], Eiichi N. Kodama*[1,5,9]

1. Department of Infectious Diseases, Tohoku University Graduate School of Medicine, 2-1 Seiryo-machi, Aoba-ku, Sendai, Miyagi 980-8575, Japan
2. Department of Bioinformatics and Integrative Omics, National Institute of Infectious Diseases, Japan Institute for Health Security, Musashimurayama, Tokyo 208-0011, Japan
3. Laboratory of Medicinal Chemistry, Kyoto Pharmaceutical University, 1 Misasagi-Shichono-cho, Yamashina-ku, Kyoto 607-8412, Japan
4. Graduate School of Pharmaceutical Sciences, Kyoto University, 46-29 Yoshida-Shimo-Adachi-cho, Sakyo-ku, Kyoto 606-8501, Japan
5. Division of Infectious Diseases, International Research Institute of Disaster Science, Tohoku University, 2-1 Seiryo-machi, Aoba-ku, Sendai, Miyagi 980-8575, Japan
6. Department of Pediatrics, School of Medicine, Fukushima Medical University, 1 Hikariga-oka, Fukushima City, Fukushima 960-1295, Japan
7. Division of Biomedical Measurements and Diagnostics, Graduate School of Biomedical Engineering, Tohoku University, 2-1 Seiryo-machi, Aoba-ku, Sendai, Miyagi 980-8575, Japan
8. Center for ViroScience and Cure, Laboratory of Biochemical Pharmacology, Department of Pediatrics, Emory University School of Medicine, Atlanta, GA 30322, USA
9. Tohoku Medical Megabank Organization, Tohoku University, 2-1 Seiryo-machi, Aoba-ku, Sendai, Miyagi 980-8575, Japan

Table S1. Inhibitory effects of the peptides for HR1–HR2 bindings determined by SPICA

Compound and peptides	IC_{50} (nM) [pIC_{50}±SD]	
	Long	CH18537
$Vir^{RS}2$	0.012 [10.9 ± 1.3]	0.0011 [12.0 ± 0.4]
$Vir^{RS}6$	0.076 [10.1 ± 0.7]	0.016 [10.8 ± 0.6]
$Vir^{RS}8$	0.12 [9.9 ± 0.6]	0.016 [10.8 ± 0.5]
$Vir^{RS}12$	0.24 [9.6 ± 0.6]	0.12 [9.9 ± 0.6]

The effects were estimated by the log-transformed half maximal inhibitory concentration (pIC_{50}).

Table S2. Interactions between amino acids in HR1 and 2 shown by protein crystallography

Construction of HR2	Amino acid of HR2	Amino acid of HR1	Binding
Disorder	Leucine 481	Valine 207	Hydrophobic
	Leucine 481	Isoleucine 206	Hydrophobic
	Leucine 481	Asparagine 208	Hydrogen*
	Leucine 481	Leucine 204	Hydrophobic
	Leucine 481	Leucine 203	Hydrophobic

The crystal structure from Protein Data Bank data, 3RRR[1], was analyzed. Single asterisk means a hydrogen binding to peptide bonds.

Table S3. Nucleotide sequence of primers for RT-PCR, sequencing and cloning

	Primer name	Sequences
RT-PCR	RT-A2-F	5’- CCCCGTCGACATGAATTATACACTCAACAATGCCAAA -3’
	RT-A2-R	5'- CCCGGATCCATGATTAGGTGCTATTTTTATTTAGTTA -3'
	RT-Long-F	5'- CCCCGTCGACATGAATTATACACTCAACAATACCAAA -3'
	RT-Long-R	5'- CCCGGATCCTGACTGGTGTGCTTCTGGCCTTA -3'
	RT-CH18537-F	5'- CCCCGTCGACATGAACTACACAATCAATACCACAA -3'
	RT-CH18537-R	5'- CCCGGATCCTGCTGAATGCAATATTATTGATTCCA -3'
For Sequence reaction	HR2-F1	5'- CACGATGCGTCCGGCGTAGAGGATCGA -3'
	HR2-F2	5'- GGACTACGTCACCGACTCGGCTGCA -3'
	HR2-F3	5'- AGAGGATTCACAAGAACATACCGGCA -3'
	HR2-R1	5'- GCGCGTCCCATTCGCCAATCCGGATA -3'
	HR2-R2	5'- CTTTGGTATTTAGCGCCTGGGTGA -3'
	T7 pro	5'- TAATACGACTCACTATAGGG -3'
	T7 term	5'- ATGCTAGTTATTGCTCAGCGG-3'
	HR1-F1	5'- GACAATTAATCATCGGCTCGTATA -3'
	HR1-F2	5'- CCCGCCAAAAACCTGGGAAGAGA -3'
	HR1-R1	5'- TCCGCTCCCGGCGGATTTGTCCTA -3'
	HR1-R2	5'- TCGTAAGACTTCAGCGCTACGGCA -3'
For Cloning	ALP-F	5'- GACGGATCCCCGGACACCAGAAATGCCTGTTCTGGAA -3'
	ALP-R	5'- CCCAAGCTTACCACCACCACCTTTCAGCCCCAGAGCGGCTTTCATGGTG -3'
	A2-HR1-F	5'- CCCGTCGACGCCAGTGGCGTTGCTGTATCTAA -3'
	A2-HR1-R	5'- CCCGGATCCTTAAATAGGTAACAATTGTTTATCTA -3'
	A2-HR2-F	5'- CCCAAGCTTATAATAAATTTCTATGACCCATTAGTA -3'
	A2-HR2-R	5'- CCCCTCGAGTTATGTGGTGGATTTAACAGCATTTACA -3'
	A2-HR2-R-C45	5'- CCCCTCGAGTTATTTAACAGCATTTACATTATGTAATAATTCA -3'
	A2-HR2-R-Vir^{RS}1	5'- CCCCTCGAGTTATACATTATGTAATAATTCATCGGATTTA -3'
	A2-HR2-R-Vir^{RS}2	5'- GGGGCTCGAGTTATAATAATTCATCGGATTTACGAATAAATGC -3'
	A2-HR2-R-Vir^{RS}3	5'- CCCCTCGAGTTAGGATTTACGAATAAATGCTAGGCTCTGGTTA -3'
	A2-HR2-R-Vir^{RS}4	5'- GGGGCTCGAGTTAAATAAATGCTAGGCTCTGGTTAATCTTC -3'
	A2-HR2-R-Vir^{RS}4$_{LAFI}$	5'- CCCCTCGAGTTATGCTGCTGCTGCGCTCTGGTTAATCTTCTCGTTGACTTGA -3'
	A2-HR2-R-Vir^{RS}5	5'- CCCCTCGAGTTAGCTCTGGTTAATCTTCTCGTTGACTTGA -3'
	A2-HR2-F-C45	5'- CCCCAAGCTTAATTTCTATGACCCATTAGTATTCCCCTC -3'
	A2-HR2-F-Vir^{RS}6	5'- CCCAAGCTTGACCCATTAGTATTCCCCTCTGA -3'
	A2-HR2-F-Vir^{RS}7	5'- CCCAAGCTTGTATTCCCCTCTGATGAATTTGATGCA -3'

A2-HR2-F-Vir^{RS}8	5'- CCCAAGCTTTTCCCCTCTGATGAATTTGATGCA -3'
A2-HR2-F-Vir^{RS}9	5'- CCCAAGCTTGATGAATTTGATGCATCAATATCTC -3'
A2-HR2-F-Vir^{RS}9$_{DEF}$	5'- CCCAAGCTTGCAGCAGCAGATGCATCAATATCTCAAGTCAACGA -3'
A2-HR2-F-Vir^{RS}10	5'- CCCAAGCTTGATGCATCAATATCTCAAGTCAACGA -3'
A2-HR2-F-Vir^{RS}11	5'- CCCAAGCTTTCTCAAGTCAACGAGAAGATTAACCA -3'
A2-HR2-F-Vir^{RS}11$_{SQVN}$-1	5'- GCAGCAGCAGCAGAGAAGATTAACCAGAGCCTAGCA -3'
A2-HR2-F-Vir^{RS}11$_{SQVN}$-2	5'- CCCAAGCTTGCAGCAGCAGCAGAGAAGATTAACCAGA -3'
A2-HR2-F-Vir^{RS}11$_{EKI}$-1	5'- TCTCAAGTCAACGCAGCAGCAAACCAGAGCCTAGCATTTATTCGTA -3'
A2-HR2-F-Vir^{RS}11$_{EKI}$-2	5'- CCCAAGCTTTCTCAAGTCAACGCAGCAGCAAACCA -3'
A2-HR2-F-Vir^{RS}11$_{NQS}$-1	5'- GAGAAGATTGCAGCAGCACTAGCATTTATTCGTAAATCCGATGA -3'
A2-HR2-F-Vir^{RS}11$_{NQS}$-2	5'- CCCAAGCTTTCTCAAGTCAACGAGAAGATTGCAGCAGCACTAGCATTTA -3'
Long-HR1-F	5'- CCCGTCGACGCTGTATCTAAGGTCCTGCACTTAGAA -3'
Long-HR1-R	5'- CCCGGATCCTTACTTATTCACAATAGGTAACAA -3'
Long-HR2-F	5'- CCCAAGCTTAATTTCTATGACCCATTAGTA -3'
Long-HR2-R	5'- CCCCTCGAGTTATTTACCAGCATTTACATTATGTAATA -3'
CH18537-HR1-F	5'- CCCGTCGACGGATCTGCAATAGCAAGTGGTATA -3'
CH18537-HR1-R	5'- CCCGGATCCTTATAATCGGTTATTTATGTAATTCTTGA -3'
CH18537-HR2-F	5'- CCCAAGCTTCCTTCTGATGAGTTTGATGCATCAATA -3'
CH18537-HR2-R	5'- CCCCTCGAGTTAAATATTTGTAGTAGATTTGCCAGTA -3'

Underlined and orange letters indicate restriction enzyme sites and a four-glycine in a linker, respectively.

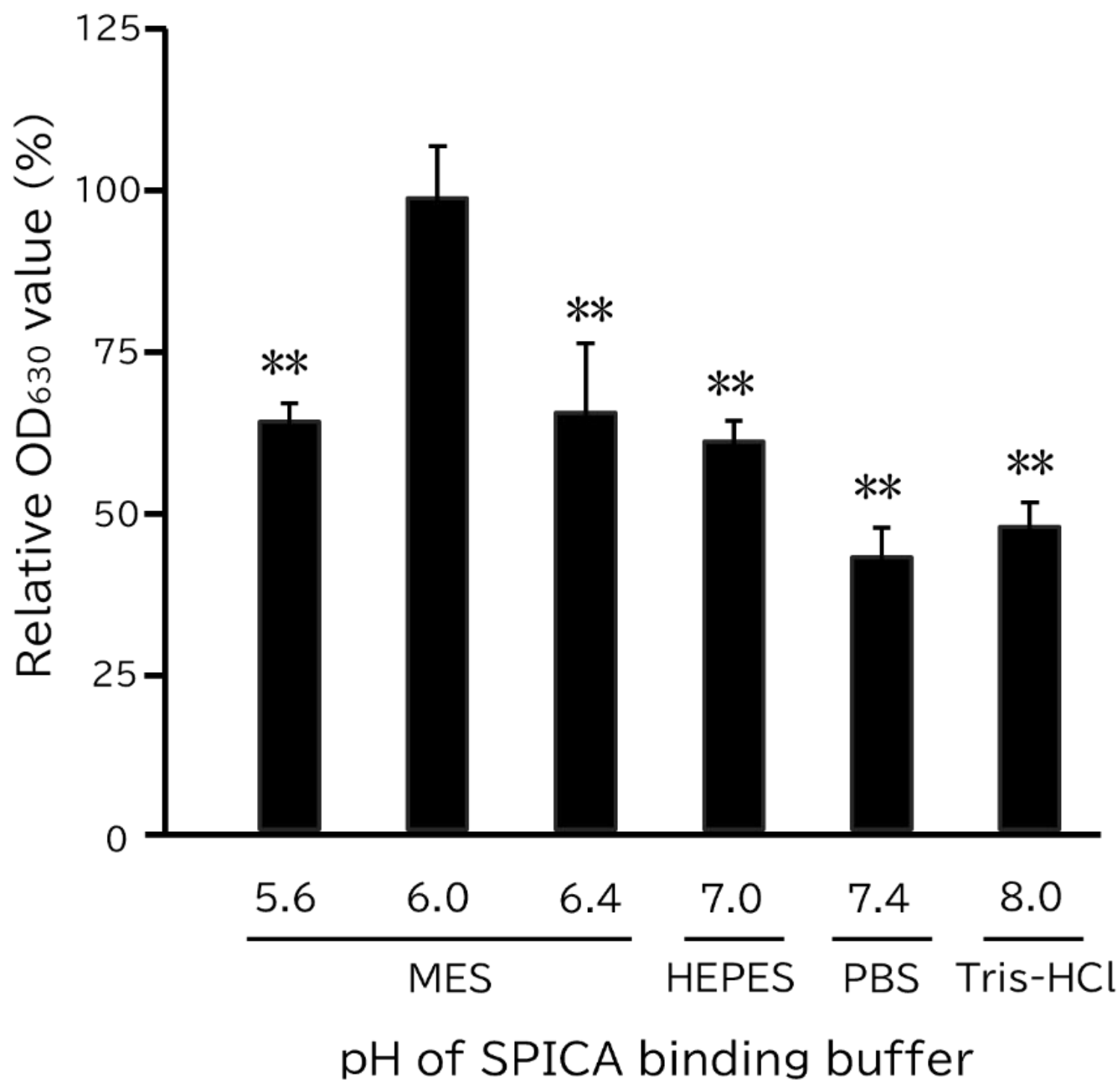

Figure S1. pH optimization for the interaction of ALP-HR2 (10 nM) with MBP-HR1 (50 nM) in SPICA

The pH value of the SPICA buffer influenced the HR1–HR2 interaction. To examine the optimal reaction solution range, the buffers (100 mM NaCl and 2 mM $MgCl_2$) were adjusted with pH 5.6, 6.0 and 6.4, or 7.0, or 7.4, or 8.0. The activity of ALP at different pH values was calculated relative to the ALP activity at pH 6.0. Error bars represent 95% confidence intervals. Double asterisks mean p value < 0.01 compared with the ALP activity at pH 6.0 by Welch's t test.

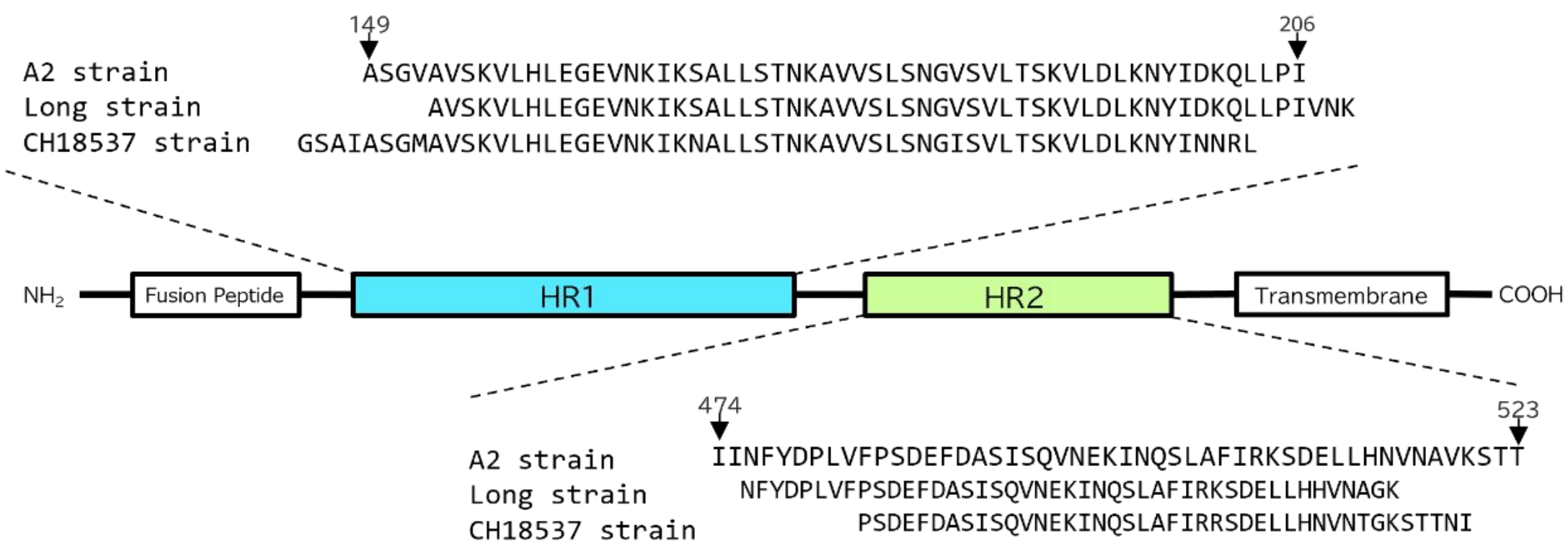

Figure S2. Amino acid regions of viral F1 protein

Positions of fusion peptide HR1, HR2, and transmembrane domain in F1 are depicted. HR1 and HR2 sequences were reported in for A2 strain[2] , Long strain[3,4], and CH18537 strain[5]. For the SPICA, the sequences of viruses are confirmed and used.

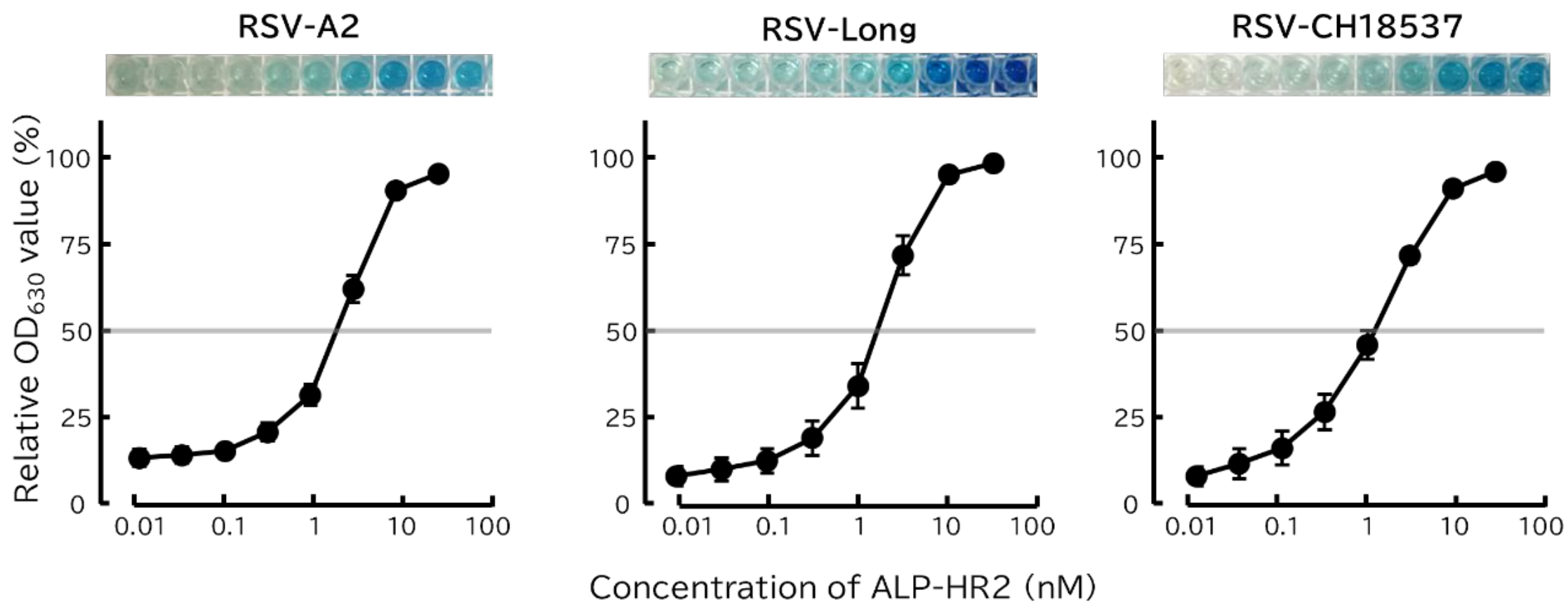

Figure S3. The HR1–HR2 interaction of three strains

ALP activity determined by SPICA represents HR1–HR2 interactions for subgroup A (RSV-A2, -Long) and subgroup B (RSV-CH18537). The interactions were estimated by the log-transformed dissociation constants (pK_d). K_d [pK_d±SD] were 2.1 nM [8.7 ± 0.2], 2.1 nM [8.7 ± 0.0], and 1.4 nM [8.9 ± 0.1] for A2, Long, and CH18537 strains, respectively. Error bars represent 95% confidence intervals.

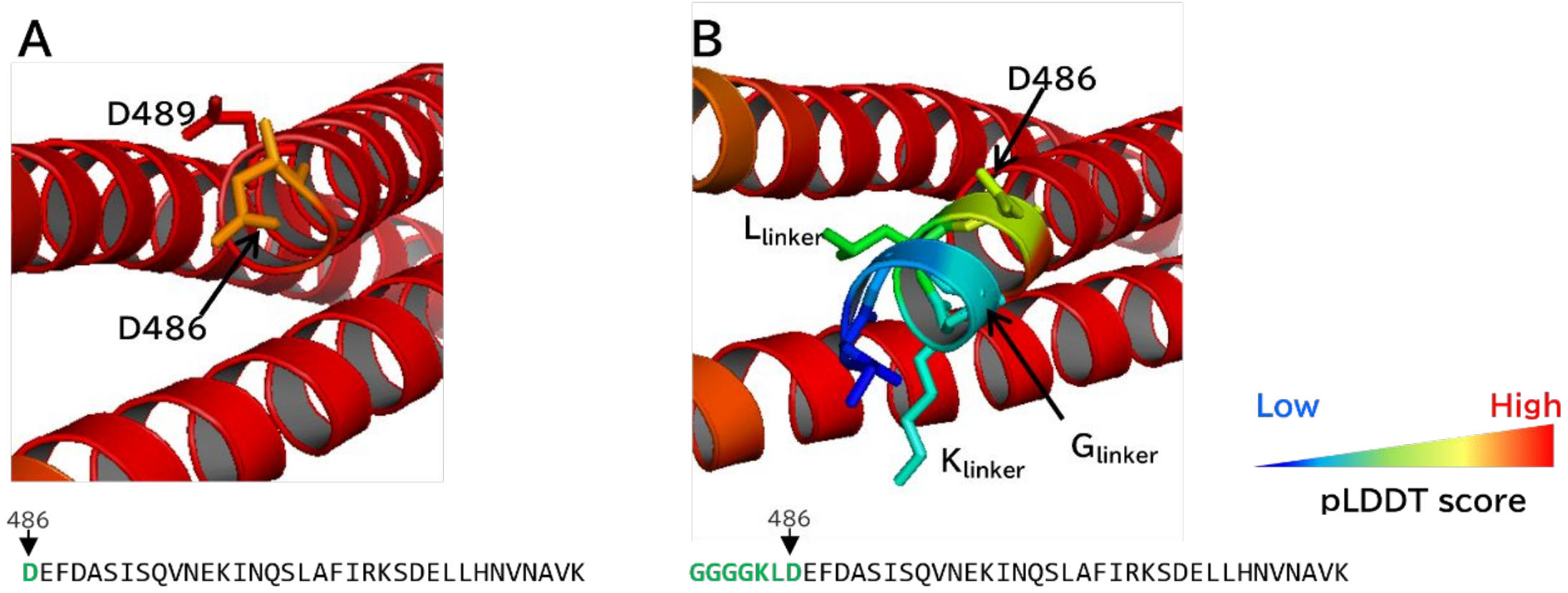

Figure S4. Predicted structure of N-terminal Vir^{RS}9 and HR1 by AlphaFold2

Interaction mode of two helical HR1s and one Vir^{RS}9 are shown. According to the pLDDT score, D486 (pLDDT = 84) was predicted as a static part of N-terminal α-helix region of Vir^{RS}9 **(A)**, but ALP-Vir^{RS}9 introduced a linker (N-terminal side) showed that D486 (pLDDT = 68) indicated low pLDDT score compared to Vir^{RS}9 without the linker **(B)**. The predicted structures of 6HB containing HR1 with either Vir^{RS}9 or ALP-Vir^{RS}9 exhibited pLDDT values greater than 90 (92 and 91, respectively).

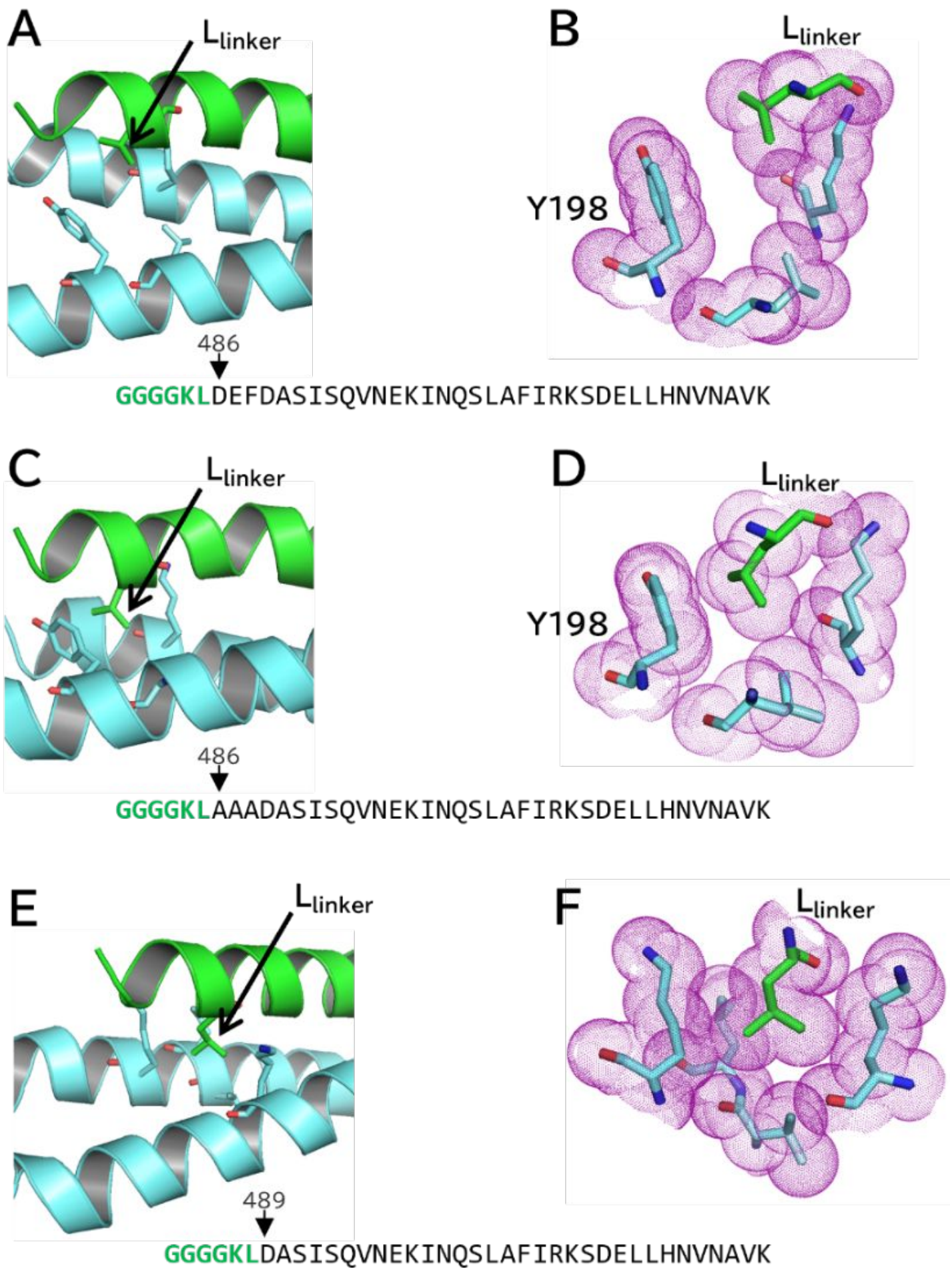

Figure S5. Predicted structure of the linkers of ALP-Vir^{RS}9, -Vir^{RS}9$_{DEF}$, -Vir^{RS}10 with HR1 by AlphaFold2

Interaction mode of two helical HR1s (cyan) and one ALP-Vir^{RS}9 **(A, B)**, -Vir^{RS}9$_{DEF}$ **(C, D)**, -Vir^{RS}10 **(E, F)** (green) with the linker (N-terminal side) are shown. Leucine in the linker of ALP-Vir^{RS}9 was predicted in outside the hydrophobic pocket formed by the HR1 **(A, B)**. In contrast, Leucine in the linker (L_{linker}) of ALP-Vir^{RS}9$_{DEF}$ and ALP-Vir^{RS}10 was predicted to be in the hydrophobic pocket **(C to F)**. The predicted structures of 6HB containing HR1 with ALP-Vir^{RS}9, -Vir^{RS}9$_{DEF}$ or -Vir^{RS}10 exhibited pLDDT values greater than 90 (91, 90 and 92, respectively). pLDDT values of L_{linker} in ALP-Vir^{RS}9, -Vir^{RS}9$_{DEF}$ and -Vir^{RS}10 are 56, 69 and 94, respectively. The composition atoms in **(B), (D) and (F)** are displayed in van der Waals volume.

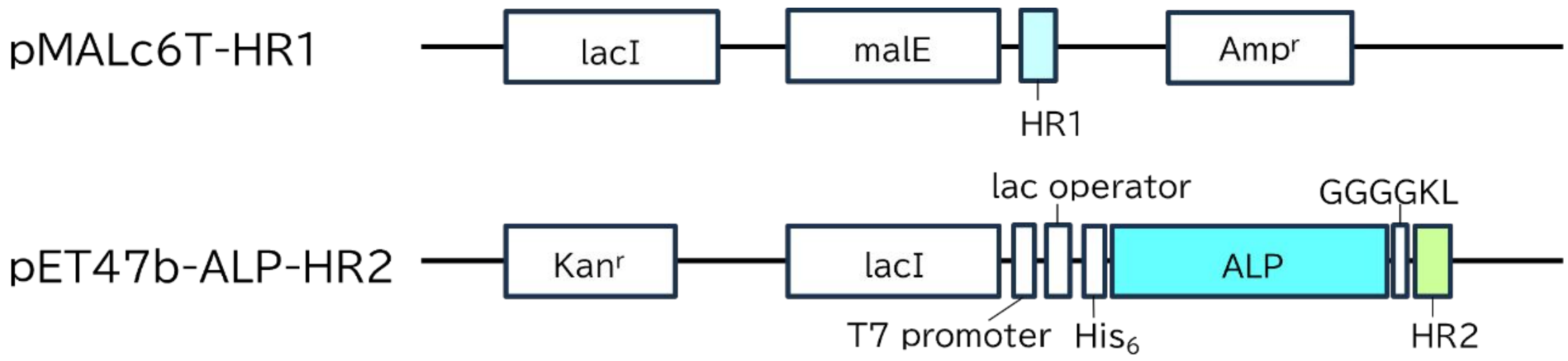

Figure S6. Construction of plasmids

The amplified HR1 fragments were inserted into the pMAL-c6T vector via SalI/BamHI sites, to generate a maltose binding protein (MBP) fused HR1. The DNA fragment coding for alkaline phosphatase (ALP) coding region (22-471 amino acids) without the secretory signal (1-21 amino acids) was amplified by PCR from *E. coli*, JM109 genome. The ALP DNA fragment fused DNA coding a four-glycine at the 3'-terminus was generated by PCR. The ALP-four-glycine DNA was cloned into the pET47b vector via BamHI/HindIII sites. The various length of cDNA fragments derived from the HR2 region were amplified by RT-PCR from Hep-2 cells infected by RSV-A2, -Long, and -CH18537. The amplified cDNAs were inserted into the pET47b-ALP vector via HindIII/XhoI sites. The ALP-HR2 were designed to be expressed with a hexa-histidine tag (His$_6$) fused to the N-terminal amino acid of ALP. As a result, the cDNA coding ALP and mHR2 are connected by a linker sequence coding four-glycine and lysine, leucine derived from HindIII site (GGGGKL). lacI and malE means the lac repressor and the maltose binding protein, respectively. Amp^r and Kan^r means the *ampicillin resistance* and kanamycin resistance, respectively.

REFERENCES

(1) McLellan, JS.; Yang, Y.; Graham, BS.; Kwong. PD. Structure of Respiratory Syncytial Virus Fusion Glycoprotein in the Postfusion Conformation Reveals Preservation of Neutralizing Epitopes. *J. Virol.* **2011,** 85, 7788-7796.

(2) Lawless-Delmedico, MK.; Sista, P.; Sen. R.; Moore, NC.; Antczak, JB.; White, JM.; Greene, RJ.; Leanza, KC.; Matthews, TJ.; Lambert, DM. Heptad-repeat regions of respiratory syncytial virus F1 protein form a six-membered coiled-coil complex. *Biochemistry* **2000,** 39, 11684-11695.

(3) Wang, E.; Sun, X.; Qian, Y.; Zhao, L.; Tien, P.; Gao, GF. Both heptad repeats of human respiratory syncytial virus fusion protein are potent inhibitors of viral fusion. *Biochem. Biophys. Res. Commun.* **2003,** 302, 469-475.

(4) Zhao, X.; Singh, M.; Malashkevich, VN.; Kim, PS. Structural characterization of the human respiratory syncytial virus fusion protein core. *Proc. Natl. Acad. Sci. U S A* **2000,** 97, 14172-14177.

(5) Matthews, JM.; Young, TF.; Tucker, SP.; Mackay, JP. The core of the respiratory syncytial virus fusion protein is a trimeric coiled coil. *J. Virol.* **2000,** 74, 5911-5920.
